# Cardiac Adaptation and Preferential Streaming in Fetuses with Multiple Nuchal Cords

**DOI:** 10.3390/diagnostics14010077

**Published:** 2023-12-28

**Authors:** Julia Murlewska, Sławomir Witkowski, Łucja Biały, Maria Respondek-Liberska, Maciej Słodki, Iwona Strzelecka

**Affiliations:** 1 Department of Prenatal Cardiology, Polish Mother’s Memorial Hospital Research Institute, 93-338 Lodz, Poland; 2 Department of Diagnoses and Prevention of Fetal Malformations of Medical, University of Lodz, 90-136 Lodz, Poland; 3 Collegium Medicum, Masovian University in Plock, 09-402 Plock, Poland

**Keywords:** fetal echocardiography, umbilical cord, nuchal cord, cardiac disproportion, cardiac remodeling, prenatal diagnosis, pediatric cardiology

## Abstract

The echocardiographic monitoring of a fetus with multiple nuchal cords around the fetal neck is important as it may result in cardiac remodeling and preferential streaming, thus affecting the condition of the fetus. The main aim of our study was to assess whether the collision of the umbilical cord around the fetal neck can lead to discrepancies in the size of the pulmonary artery and the aorta in the three-vessel view and to an increase in the size of the heart, which may result from the compression of the carotid arteries caused by the umbilical cord wrapping around the fetal neck. A total number of 854 patients were included in this study and divided into three groups. Group A (control group) included 716 fetuses (84%) without the umbilical cord around the fetal neck. Group B (study group B) included 102 fetuses (12%) with one coil of the umbilical cord around the fetal neck. Group C (study group C) included 32 fetuses (4%) with two coils of the umbilical cord around the fetal neck. The range of the gestational age of the patients considered for this study was 27–40.2 weeks based on the ultrasound biometry and was not statistically different between the analyzed groups A, B and C (*p* > 0.05). The Pa/Ao index was calculated by dividing the value of the width of the pulmonary artery (in mm) to the width of the aorta (in mm) measured in the ultrasound three-vessel view. We found that fetuses that the fetuses with one and two coils of the umbilical cord around the neck showed significantly higher values of the width of the pulmonary trunk with the unchanged value of the aorta width. Therefore, we also observed significantly higher values of the ratio of the pulmonary trunk to the aorta for the fetuses wrapped with the umbilical cord around the neck compared with the control group without the umbilical cord around the neck (. Moreover, in the fetuses with one and two coils of the umbilical cord around the fetal neck, an increased amount of amniotic fluid was observed, whereas larger dimensions of CTAR in the fetuses with two coils of the umbilical cord around the neck were present (*p* < 0.05). The wrapping of the fetus with the umbilical cord around the fetal neck may induce the redistribution of blood flow, leading to fetal heart enlargement and disproportion and may be the cause of polyhydramnios.

## 1. Introduction

The disproportion of the fetal heart in the four-chamber view and three-vessel view with the dominance of the right side of the fetal heart in the third trimester of pregnancy may be a variant of the norm; however, aortic coarctation must be excluded first. The main pulmonary artery (MPA) dilatation in the three-vessel view can also be observed in the course of the fetal heart defects such as isolated pulmonary valve stenosis with the poststenotic dilatation of the main pulmonary artery or Tetralogy of Fallot with absent pulmonary valve syndrome [1].

The issue of the wrapping of the umbilical cord around the fetal neck has been analyzed in the literature mainly in the context of the effect on the perinatal outcomes [2,3,4,5]; however, very few data have been published on the effect of umbilical cord wrapping on the prenatal echosonographic parameters of the fetus [6], which are exponents of fetal blood circulation assessment. Due to the use of innovative echocardiographic technologies, modern prenatal cardiology is currently focused on functional analysis and functional echocardiographic monitoring of the fetal heart. In response to unfavorable conditions in utero or the potential compression of the umbilical cord wrapped multiple times around the fetal neck, the fetal heart may show functional adaptive remodeling or dysfunctional abnormalities, which are also observed in the course of intrauterine infections, pregnancy-induced hypertension, preeclampsia or gestational diabetes [7]. With reference to the work by Wieckowska, K., et al. [8], who presented a comparative analysis of 10 cases of fetuses with the umbilical cord wrapped around the neck, the aim of our study was to assess whether the collision of the umbilical cord around the fetal neck can lead to discrepancies in the size of the pulmonary artery and the aorta in the three-vessel view and to an increase in the size of the heart, which may result from the compression of the carotid arteries due to the umbilical cord wrapping around the fetal neck.

## 2. Materials and Methods

This was a single-center analysis of a group of fetuses who had fetal ultrasound examinations at the tertiary center from January 2018 to March 2023. All the information about the fetuses was collected from the database of our unit. All the healthy fetuses were labelled with normal biometry, normal heart anatomy and presented no extracardiac malformations.

**Figure 1:**
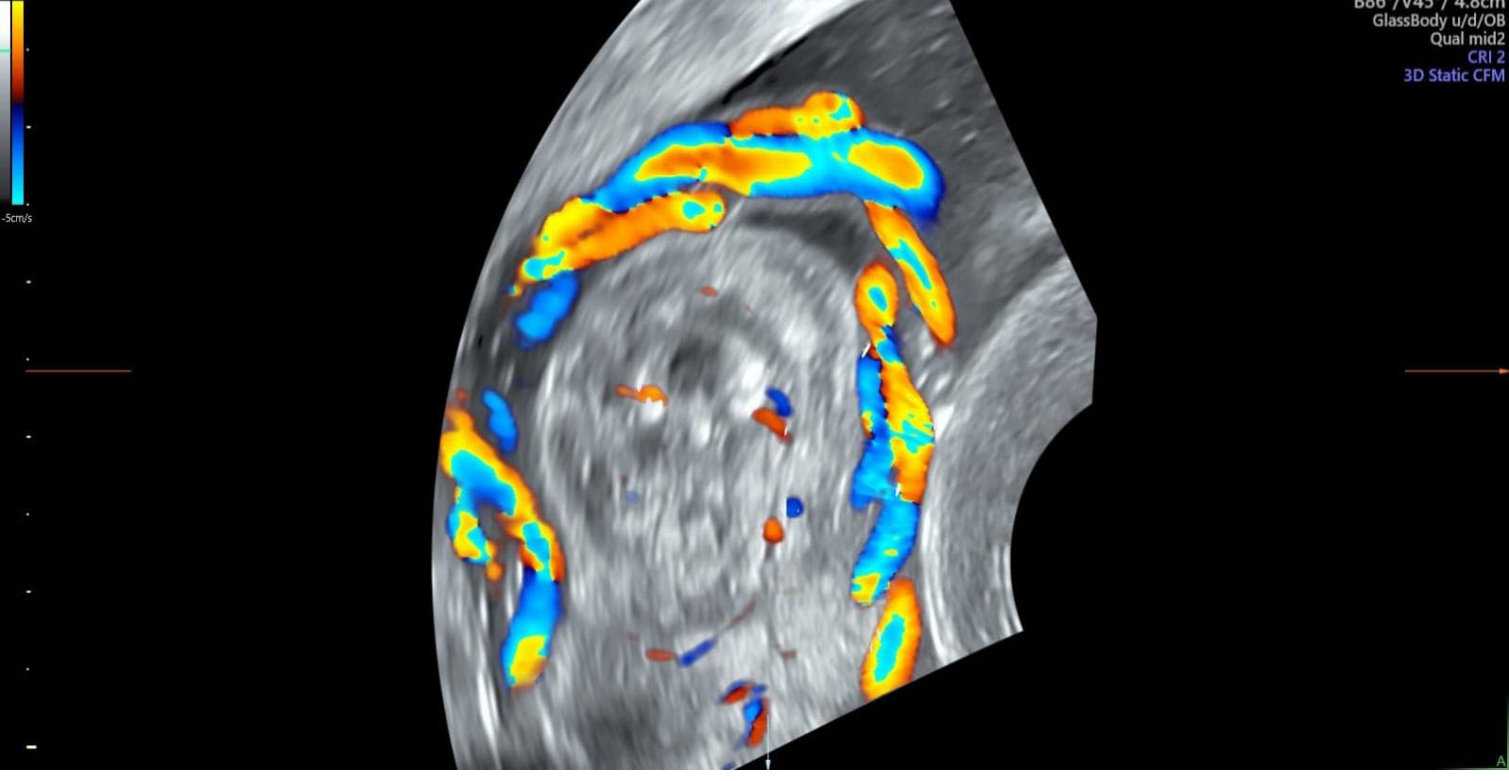
Ultrasound entanglement from right to left of umbilical cord around the fetal neck. Blood flow characteristics: the right half ring has two blue and one red flows, while the left half ring has two red and one blue flows, which shows that the blood flow is anticlockwise and suggests that the umbilical cord on the neck coils from left to right.

**Figure 2:**
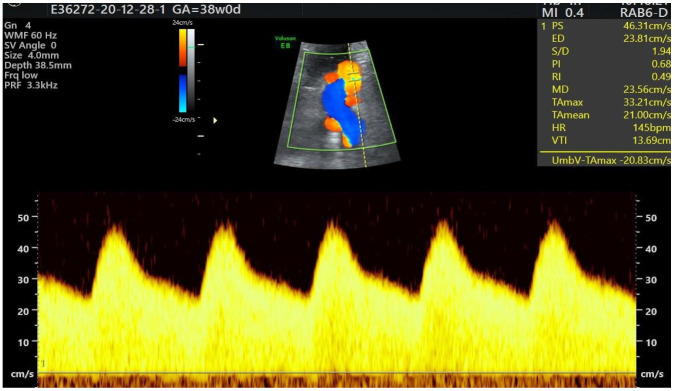
Normal spectrum of umbilical flow at 38 weeks of gestation in a fetus with one coil of the umbilical cord around the neck.

**Figure 3:**
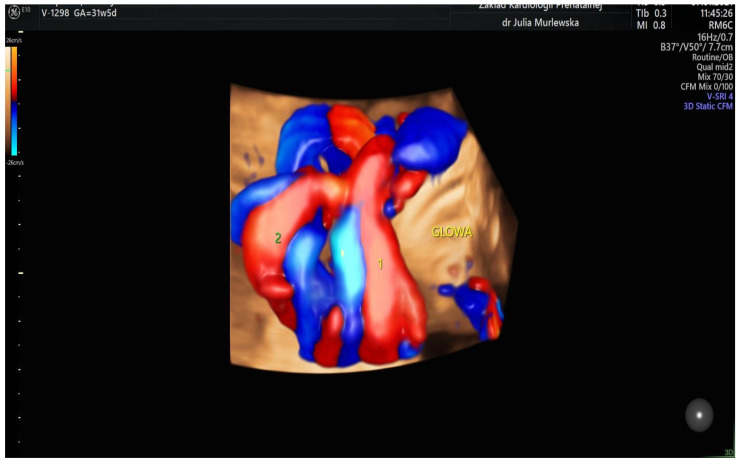
A fetus with two coils of the umbilical cord around the fetal neck. The entanglement angle is 360° and the blood flow in both of the coils is upwards, i.e., clockwise.

**Figure 4:**
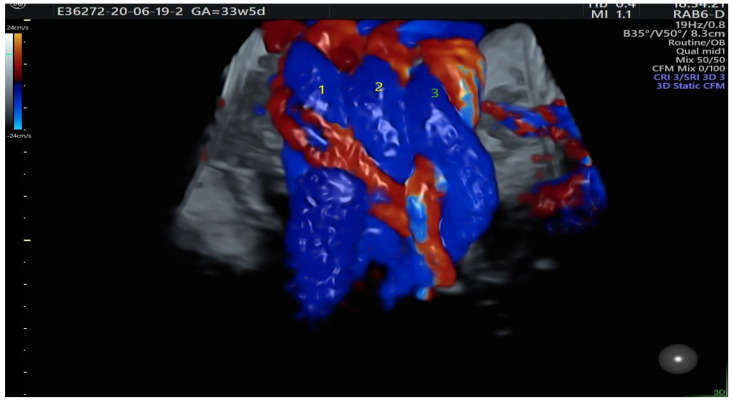
A fetus with three coils of the umbilical cord around the fetal neck. The entanglement angle is 360° and the blood flow is downwards in all of them, i.e., anticlockwise.

A total of 3791 patients were considered for this study. However, 2937 fetuses were excluded from this study due to their gestational age of under 27 weeks and the lack of some crucial information such as fetal heart size, width of the pulmonary artery or the aorta or chronic maternal diseases. All measurements were performed by two experienced prenatal echocardiographers who, in addition to the echocardiographic assessment of the fetal heart, evaluated the status of the course of the umbilical cord wrapped around the fetal neck. Only cases with 360-degree entanglement angle were considered for this study. Figure 1, Figure 2, Figure 3 and Figure 4 show the observation and characteristics of the fetal umbilical cord wrapping, blood flow measurements in the umbilical artery, as well as multiple cord wrapping schemes such as the double and triple wrapping of the umbilical cord around the fetal neck with the use of the three-dimensional power Doppler imaging. The analyzed 854 patients were divided into three groups. In group A (control group), *n* = 716 fetuses (84%) without the umbilical cord around the fetal neck. In group B (study group B), *n* = 102 fetuses (12%) with one coil of the umbilical cord around the fetal neck. In group C (study group C), *n* = 32 fetuses (4%) with two coils of the umbilical cord around the fetal neck. The gestational age of the patients considered for this study ranged from 27 weeks to 40.2 weeks, according to the ultrasound biometry, and there were no statistical differences between the analyzed groups A, B and C (*p* > 0.05). The Pa/Ao index was calculated by dividing the value of the width of the pulmonary artery (in mm) by the width of the aorta (in mm) measured in the ultrasound three-vessel view.

### 2.1. Exclusions and Final Sample Number

Within the studied group of 854 fetuses, there were 42 cases of fetuses with a four-chamber disproportion of the heart (6 cases of fetuses with 2 coils of the umbilical cord around the neck, 9 cases of fetuses with 1 coil of the umbilical cord around the neck and 27 cases of fetuses without the umbilical cord around the neck) and 9 cases of fetuses with single umbilical artery (SUA). However, both disproportion and SUA did not significantly affect the results of our study. Four cases of fetuses with the three coils of the umbilical cord around the neck were excluded from the analysis due to a small sample size, which is not a representative group for drawing conclusions. Finally, a total number of 854 patients with complete data on the number of umbilical cords around the neck, such as estimated fetal weight (EFW), amniotic fluid index (AFI), single umbilical artery (SUA), pulsatility index for the umbilical artery (UmbA PI), pulsatility index for the middle cerebral artery (MCA PI), width of the pulmonary artery (Pa) and the aorta (Ao) as well as cardiothoracic ratio (CTAR), were included in this study.

### 2.2. Statistical Analysis

Statistical analysis was performed using TIBCO Statistica 13.3 and Microsoft Excel 365 software. Scatter graphs of the fetal Pa/Ao index were created for groups A, B and C. The data were not normally distributed. The Mann–Whitney U test was used for preparing this analysis. A box plot with minimal, maximal and median values was created for a comparison of the width of the pulmonary artery in group A with group B and group C. A *p*-value of < 0.05 was considered statistically significant.

## 3. Results

**Figure 5:**
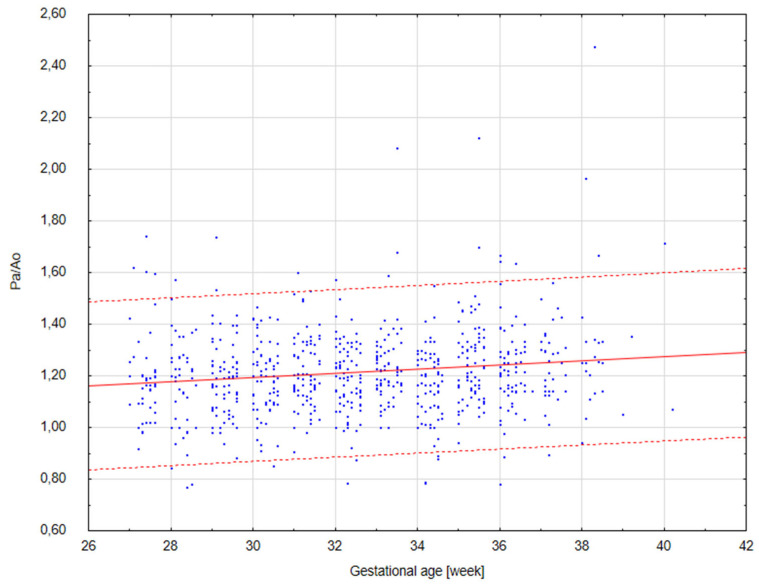
A scatter graph of fetuses from group A (*n* = 716) without the umbilical cord around the neck based on the relationship between the gestational age (in weeks) and the Pa/Ao index.

**Figure 6:**
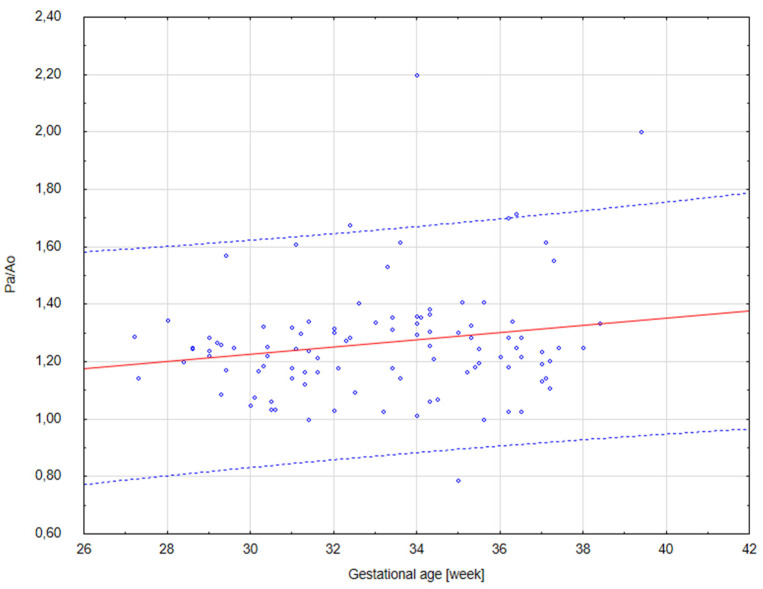
A scatter graph of fetuses from group B (*n* = 102) with one coil of the umbilical cord around the neck based on the relationship between the gestational age (in weeks) and the Pa/Ao index.

**Figure 7:**
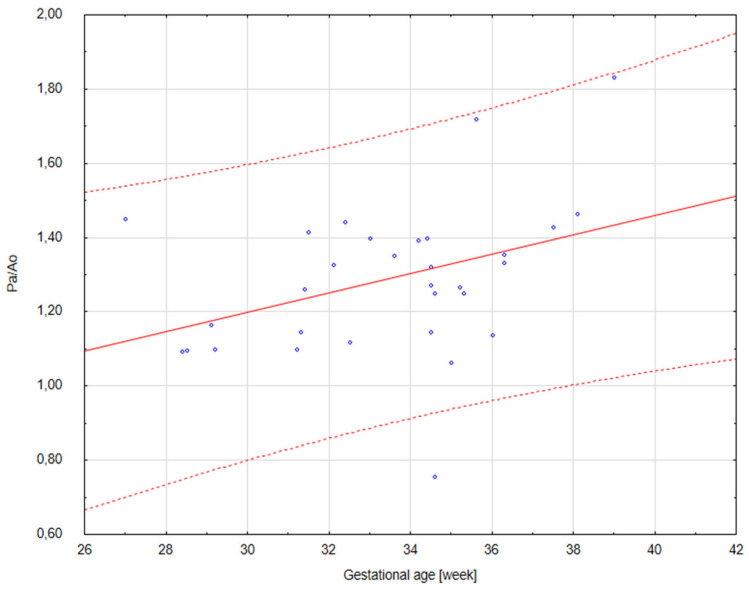
A scatter graph of fetuses from group C (*n* = 32) with two coils of the umbilical cord around the neck based on the relationship between the gestational age (in weeks) and the Pa/Ao index.

**Figure 8:**
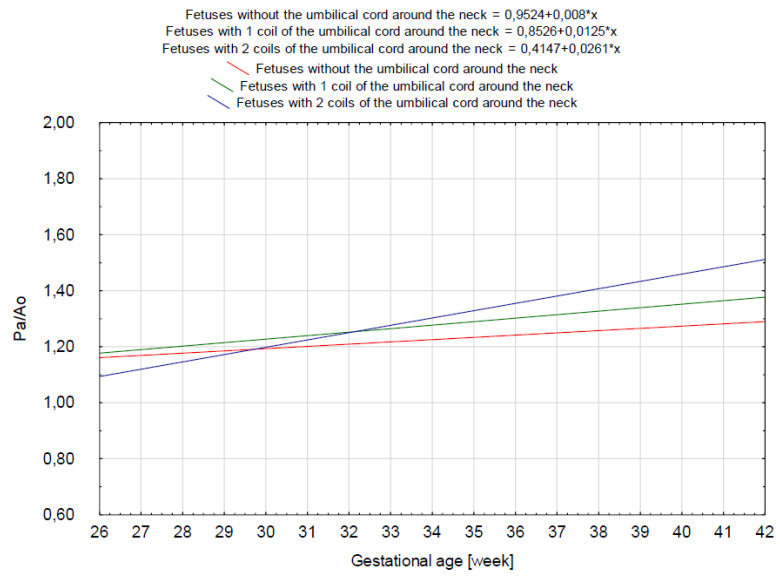
A trend line for 854 fetuses from group A, B and C compared together in one graph in relation to the gestational age (in weeks) and the Pa/Ao index.

**Figure 9:**
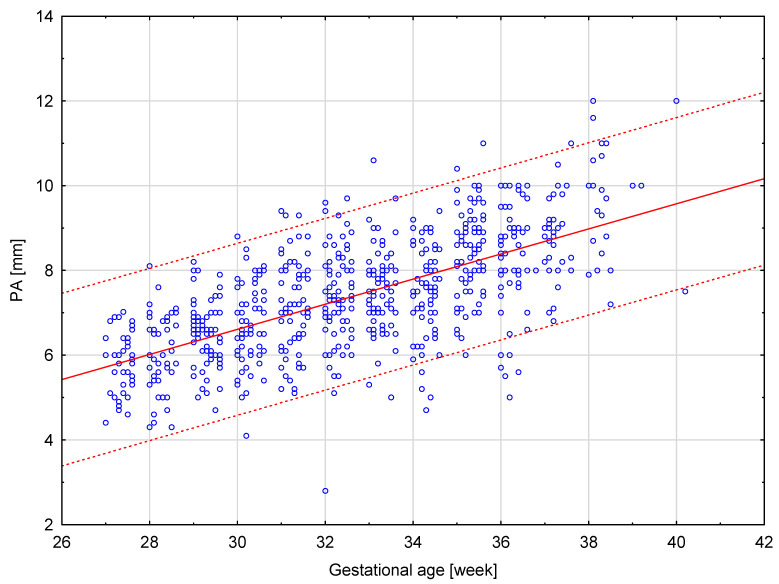
A normal range of PA (mm) in fetuses without the umbilical cord around the neck.

**Figure 10:**
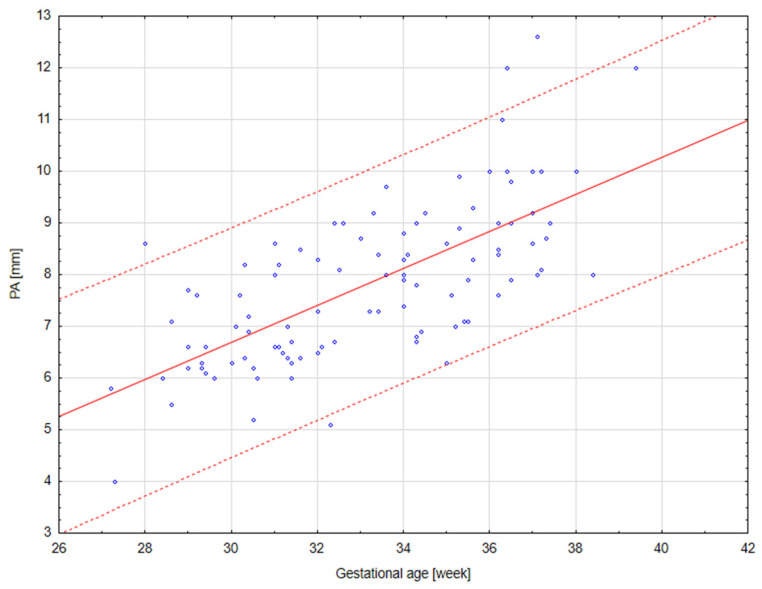
A normal range of PA (mm) in fetuses with one coil of the umbilical cord around the neck.

**Figure 11:**
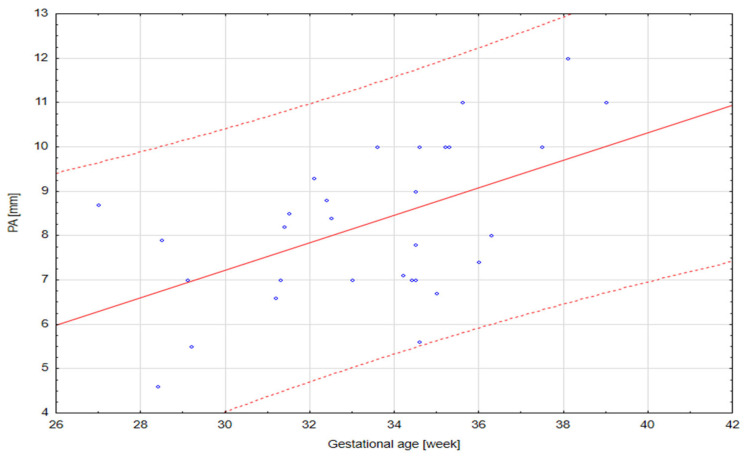
A normal range of PA (mm) in fetuses with two coils of the umbilical cord around the neck.

**Figure 12:**
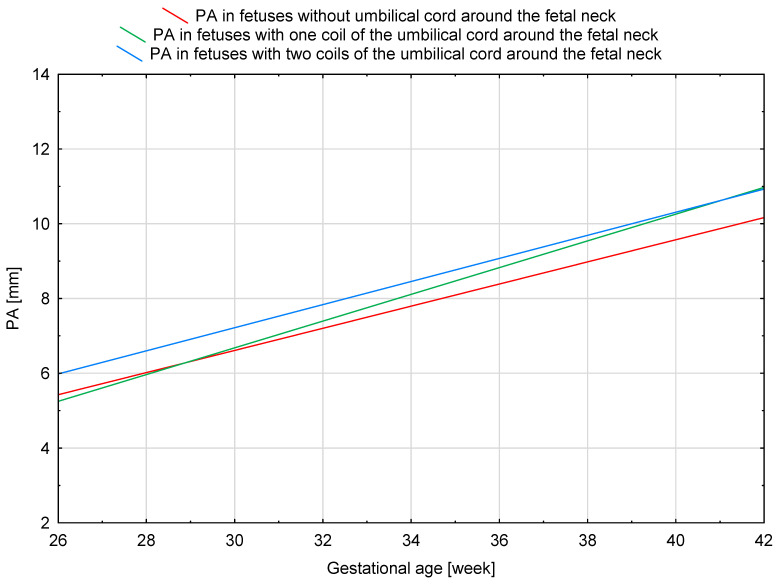
The mean values of PA (mm) in fetuses from group A, B and C.

The results of our analysis are presented in Table 1, Table 2 and Table 3. The UmbA PI, MCA PI and EFW in all the groups were not affected (*p* > 0.05). There are no statistically significant differences in PA, Ao and the Pa/Ao ratio between group B and group C (*p* > 0.05). There was a statistically significant correlation between the measurement of Pa and the gestational age in all the groups (*p* < 0.05). We found that fetuses with one and two coils of the umbilical cord around the neck showed significantly higher values of the width of the pulmonary trunk with the unchanged value of the aorta width. Therefore, we also observed significantly higher values of the ratio of the pulmonary trunk to the aorta for the fetuses wrapped with the umbilical cord around the neck compared with the control group without the umbilical cord around the neck (Figure 5, Figure 6, Figure 7, Figure 8, Figure 9, Figure 10, Figure 11 and Figure 12). Moreover, in the fetuses with one and two coils of the umbilical cord around the fetal neck, an increased amount of amniotic fluid was observed, whereas larger dimensions of CTAR in the fetuses with two coils of the umbilical cord around the neck were present (*p* < 0.05) (Table 1 and Table 2).

## 4. Discussion

Disproportion between the pulmonary artery and the aorta with the Pa/Ao ratio equal to or larger than 1.6 should be an alarming sign of the possible coarctation of the aorta (CoA) [8,9,10]. If the ascending aorta has a greater diameter than the main pulmonary artery, it usually does not suggest severe congenital heart disease but requires prenatal and postnatal echocardiographic examinations [11].

Wieckowska et al. reported for the first time that the umbilical cord enlacing the neck may mimic CoA [8]. Respondek-Liberska [12] analyzed the database of 3941 fetuses and disproportion in a four-chamber view. Thirty-four fetuses with disproportion presented ‘normal heart anatomy’ and no extracardiac malformations. Gestational age at the time of diagnosis was 26–40 weeks (mean 35 weeks). Out of 34 fetuses with isolated disproportion, 15 cases (44%) did not have any clinical or postnatal ECH0 abnormalities. In nineteen cases (54%), structural or functional abnormalities were found: six neonates presented ASD secundum, four of them presented CoA, two with lung abnormalities (congenital pneumonia and congenital lung emphysema), one neonate had patent ductus arteriosus (PDA) and one had aorto-pulmonary window with PDA. In five other neonates, tricuspid insufficiency was observed and diagnosed [12].

In our analysis of fetuses with the umbilical cord around the neck, there were no cases characterized by accelerated peak velocity in aortic isthmus, suggesting aortic isthmus stenosis. We observed disproportion in a four-chamber view in the qualitative assessment in 42 cases and in 9 cases of single umbilical artery (SUA) but without any accompanying anomalies. The umbilical cord usually has two umbilical arteries and one umbilical vein and, due to the presence of the Wharton’s jelly and Hyrtle’s anastomosis, is very well protected against tension and compression caused by fetal movements and by the umbilical cord being wrapped around the fetal neck or torso [13]. If the umbilical cord has only two vessels, one artery and one vein, it does not have Hyrtle’s anastomosis, and so it can be believed that this type of umbilical cord is more susceptible to deformation. To date, it has been shown that pregnancies with SUA have had an increased risk of SGA (small for gestational age) fetal growth disorder and pregnancy-induced hypertension [14], as well as around 11% of cases presenting congenital malformations of the digestive system, mainly gastrointestinal atresia or stenosis [15]; however, the assessment of umbilical and cardiac circulation in fetuses with two-vascular umbilical cords has not yet been sufficiently documented.

Our analysis showed that in both a small subgroup of 9 fetuses with SUA and a small subgroup of 42 fetuses with a qualitative assessment of the disproportion in a four-chamber view of the heart, the results of the echocardiographic parameters of the fetuses wrapped with the umbilical cord have not been significantly affected.

Whole blood is commonly considered a non-Newtonian fluid whose viscosity is not constant and is a function of flow velocity, while according to the Hagen–Poiseuille law, blood flow is proportional to the fourth power of the vessel radius and to the pressure difference, causing the flow to be inversely proportional to blood viscosity and the length of the vessel. Therefore, it can be assumed that multiple coils of the umbilical cord around the fetus may cause the compression of the endothelium in the carotid arteries and an increase in blood pressure. Moreover, it can lead to the redistribution of blood flow (according to the law of blood stream continuity) by increasing blood pressure in order to provide fast blood supply to the areas of priority need so as to protect the fetal heart from stress, which could lead to disproportion in the width of the vessels of the upper mediastinum and four chambers of the heart with a predominance of the right side of the heart, i.e., widening of the pulmonary trunk, right ventricle, right atrium and myocardial hypertrophy, provided that the process of squeezing the fetal neck by the umbilical cord with carotid artery stenosis lasts for a long period of time. As a result, aortic coarctation in its isthmus may lead to an increase in the blood flow and in the isthmic blood pressure (according to the Bernoulli’s law) [16,17].

The explanation of the pathomechanisms of circulatory redistribution is made on the basis of the studies on fetuses of sheep subject to hypoxia. Due to the occurrence of shunts in the fetal circulation like in ductus venosus and ductus arteriosus, preferential streaming is further ensured as an adequate supply of oxygenated blood to tissues that are at risk of damage in a situation of adverse fetal conditions [18].

Our previous studies were carried out on a group of 115 fetuses from single pregnancies with physiological courses with a single nuchal cord around the fetal neck at the 15th to 40th week of pregnancy. The peak systolic velocity in MCA in the study group with single nuchal cord was significantly higher than in the control group (40.2 ± 11.5 vs. 32.5 ± 9.5; *p* = 0.003), which would support the theory of fetal circulation being redistributed to preferential organs such as the brain and fetal heart [19].

Due to the carotid chemoreflex and fetal brain sparing effect, the umbilical cord wrapped around the neck of the fetus probably causes an increase in blood flow through the cerebral vessels and leads to redistribution and an increased flow in ductus venosus and umbilical vein, which consequently leads to heart remodeling [20], i.e., enlargement of the main pulmonary artery width, right atrium and ventricle. This may mimic the coarctation of the aorta and reduced blood flow through the aorta [8,11,12,18,21].

**Figure 13:**
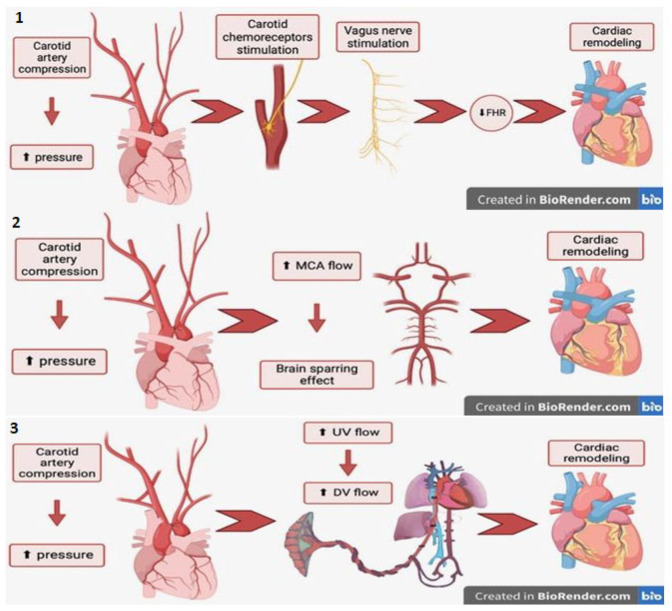
During our research, we considered three theories explaining circulatory redistribution—increased Pa/Ao ratio—in fetuses with the umbilical cord around their necks. All of them start with the compression of the fetal carotid artery by the umbilical cord, which results in an increase in pressure in the carotid artery, therefore causing the following: theory number **1**—the stimulation of carotid chemoreceptors and the vagus nerve, which may result in FHR decrease; theory number **2**—Increased flow through the middle cerebral artery (MCA), which leads to a brain-sparing effect; theory number **3**—increased flow in ductus venosus and umbilical vein [23]. Moreover, the compression of the nuchal cord (one coil vs. two coils) may affect the esophageal function, which could result in the less frequent swallowing of the amniotic fluid or the swallowing of a small amount of amniotic fluid. This may lead to polyhydramnios. As a result of nuchal cord compression, fetal hypoxia may occur. This leads to a reduction in the amniotic fluid production due to the redistribution of the fetal blood flow with a decrease in the perfusion of peripheric organs (as the kidney) and an increase in the perfusion of brain, heart, liver and adrenals [24]. In our cases, this phenomenon seems not to be the case.

There is not one correct explanation that addresses not only the wrapping of the umbilical cord around the fetal neck causes cardiac remodeling but a combination of all those three (Figure 13).

As for parameters that should be taken into consideration during the examination of the fetuses with the umbilical cord wrapped around the neck, we recommend paying strong attention to the echocardiographic parameters presented in Table 4.

Based on the results of our previous research, we know that the observation of fetal cord wrapping around the neck does not adversely affect birth outcomes [19]. In our earlier study, we found that fetuses wrapped with one coil of the umbilical cord around the neck in the third trimester of pregnancy did not require the earlier induction of pregnancy, and we did not observe an increased percentage of preterm births or the number of cesarean sections performed in this group of fetuses. In the group of fetuses wrapped with the umbilical cord (*n* = 38 (study group) vs. *n* = 77 (control group)), persistent umbilical cord wrapping during delivery was observed more often (29% in fetuses with the umbilical cord wrapping in the third trimester vs. 10% in fetuses without the umbilical cord wrapping in the third trimester), which, however, did not make the obstetric results worse [19].

There are some important clinical implications that arise from our study. This study demonstrates that the widening of the PA, and polyhydramnios may result from the wrapping of a fetus with an umbilical cord. The presence of the carotid chemoreflex and fetal brain-sparing effect, the umbilical cord wrapped around the neck of the fetus probably leads to redistribution and an increased flow in ductus venosus and umbilical vein, which consequently leads to heart remodeling [20], i.e., the enlargement of the main pulmonary artery width, right atrium and ventricle. This may mimic the coarctation of the aorta and reduced blood flow through the aorta. So far, the experience with fetal sheep studies has shown that the intermittent occlusion/clamping of the umbilical cord results in hypoxemia and acidosis. The aim of our retrospective analysis was to demonstrate the effect of multiple wrapping of the umbilical cord around the fetal neck on selected echocardiographic parameters. The results of our analysis allow us to show which of the very diverse and modern techniques should be used for the specialized monitoring of the fetus wrapped with the umbilical cord.

In the case of the 2D classic echocardiographic technique:

- HA/CA (heart area/chest area ratio);
- GSI (global sphericity index);
- 4CV analysis;
- 3VV analysis.

In the case of color Doppler:

- FHR;
- PI MCA, PS MCA;
- PI UMBA;
- Cerebroplacental ratio—CPR;
- PI DV, PS DV;
- PS UV;
- PS PA;
- PS Ao;
- PS AOI;
- PS DA, PI DA.

In the case of M-Mode:

- SF LV, SF RV, IVS;
- RV myocardial width.

In the case of speckle tracking:

- assessment of the cardiac contractility and GLS—global longitudinal sphericity index.

Fetal cardiac scanning must be performed according to the guidelines provided by us and the international guidelines for fetal heart evaluation [22].

Due to the observed changes in the heart, including PA widening and disproportion, as well as no information on a long-term prognosis of the fetuses wrapped with the umbilical cord around the neck, it is suggested to monitor such fetuses with the consideration of the above-mentioned parameters.

Our study is a continuation of our earlier research on the fetuses wrapped with the umbilical cord. Our experience has allowed us to better understand the pathological mechanisms of circulatory disorders associated with the cord compression/wrapping of the fetus. A key strength of this paper is that it presents retrospective results on a large population of fetuses, which includes 854 patients from the research and development center with the prenatal cardiology highest rank. The advantage of our study is the analysis carried out by experienced specialists, including echocardiographers, prenatal cardiologists and obstetricians, who have analyzed the issue of collision of the umbilical cord not only from the perspective of its impact on birth outcomes but also in the context of fetal circulatory status analysis, which is unusual in specialist literature.

In spite of having a large database of fetuses, a limitation of our study was the inability to observe the effect of umbilical cord wrapping over time. We cannot clearly determine what is the influence of time on the cascade of changes in the fetal heart. There also may be some maternal factors that can affect blood flow, such as abnormal velocity waveforms in the uterine arteries [25]. However, we certainly believe that in such cases it would be reasonable to monitor the fetal heart with the use of echocardiography in order to evaluate the extent of the redistribution of circulation and the evolution of changes. It is also of great importance in the context of evaluating fetal growth and in a situation of a slowdown of the fetal growth. A confirmation that a fetus is wrapped with the umbilical cord several times may allow us to find the cause of fetal growth retardation. We should also pay more attention to the amount of amniotic fluid due to the fact that our analysis demonstrates a certain relationship between the amount of amniotic fluid and the tendency of the fetal neck to be wrapped with the umbilical cord (Table 1 and Table 2). A weak part of our study may be the fact that we analyzed cross-sectional examinations, and we did not perform a longitudinal analysis to observe how long the fetus was wrapped with the umbilical cord and whether the neck of the fetus was free of the umbilical cord before delivery or not.

## 5. Conclusions

The wrapping of the fetus with the umbilical cord around the neck may induce the redistribution of blood flow, leading to fetal heart enlargement and disproportion and may be the cause of polyhydramnios.

## Figures and Tables

**Table 1:** Average values of selected parameters in group A, B and C.

Parameter	Group A	Group B	Group C
GA (biometry)	32.54 ± 3.1	33.14 ± 2.9	33.45 ± 2.9
UmbA PI	0.974 ± 0.2	1.101 ± 0.2	0.889 ± 0.3
MCA PI	1.825 ± 0.5	1.862 ± 0.5	1.788 ± 0.5
AFI	14.18 ± 3.7	15.57 ± 4.3	15.64 ± 3.6
CTAR	0.31 ± 0.03	0.31 ± 0.04	0.33 ± 0.04
EFW	2071.75 ± 653	2179.54 ± 647	2240.22 ± 677

**Table 2:** Statistical differences between groups. *p* < 0.05 is considered a statistically significant difference. * The borderline *p* = 0.04 in UmbA PI between group A and group C results from a small number of fetuses from group C and requires further studies on a larger group of fetuses in this group.

Parameter	Group A (*n* = 716) vs. Group B (*n* = 102)	Group A (*n* = 716) vs. Group C (*n* = 32)
UmbA PI	*p* > 0.05	*p* = 0.04 *
MCA PI	*p* > 0.05	*p* > 0.05
AFI	*p* < 0.05 (*p* = 0.0004)	*p* < 0.05 (*p* = 0.009)
CTAR	*p* > 0.05	*p* < 0.05 (*p =* 0.006)
EFW	*p* > 0.05	*p* > 0.05

**Table 3:** A summary of the following parameters compared between the study groups and the control group of fetuses which did not have the umbilical cord wrapped around the neck: PA—width of the pulmonary trunk; AO—width of the aorta; and the ratio of the width of the pulmonary trunk to the aorta—Pa/Aa ratio. significant dependencies are in bold.

Parameter	PA [mm]	AO [mm]	PA/AO Ratio
0 nuchal cords, *n* = 716 (group A), vs. 1 nuchal cord, *n* = 102 (group B)	**7.4 ± 1.4 vs. 7.8 ± 1.5 (*p* = 0.009)**	6.1 ± 1.1 vs. 6.2 ± 1.1 (*p* = 0.35)	**1.2 ± 0.2 vs. 1.3 ± 0.2 (*p* = 0.01)**
0 nuchal cords, *n* = 716 (group A), vs. 2 nuchal cords, *n* = 32 (group C)	**7.4 ± 1.4 vs. 8.3 ± 1.7 (*p* = 0.002)**	6.1 ± 1.1 vs. 6.4 ± 1.0 (*p* = 0.07)	**1.2 ± 0.2 vs. 1.3 ± 0.2 (*p* = 0.01)**
1 nuchal cord, *n* = 102 (group B), vs. 2 nuchal cords, *n* = 32 (group C)	7.8 ± 1.5 vs. 8.3 ± 1.7 (*p* = 0.1)	6.2 ± 1.1 vs. 6.3 ± 1.0 (*p* = 0.3)	1.3 ± 0.2 vs. 1.3 ± 0.2 (*p* = 0.2)

**Table 4:** Prenatal echocardiography recommendations for the assessment of fetuses wrapped with the umbilical cord around the neck.

2D	HA/CA (heart area/chest area ratio); GSI (global sphericity index); 4CV analysis; 3VV analysis.
Color Doppler	FHR; PI MCA, PS MCA; PI UMBA; Cerebroplacental ratio—CPR; PI DV, PS DV; PS UV; PS PA; PS Ao; PS AOI; PS DA, PI DA.
M-Mode	SF LV, SF RV, IVS; RV myocardial width.
Speckle tracking	assessment of cardiac contractility and GLS—global longitudinal sphericity index.

## Data Availability

Data available on request due to privacy reasons.
